# *In vitro* Metabolic Profiling for the Choice of Schweinfurthin Analog Development Candidate

**DOI:** 10.2174/0118723128371700251204080946

**Published:** 2026-03-13

**Authors:** Jessie L. Reed, Wesley M. Raup-Konsavage, Jeffrey D. Neighbors

**Affiliations:** 1 Department of Molecular and Precision Medicine, Penn State University College of Medicine, Hershey, PA, USA;; 2 Department of Medicine, Penn State University College of Medicine, Penn State Cancer Institute, Hershey, PA, USA

**Keywords:** Immunogenic cell death, schweinfurthin, natural products, anticancer, oxysterol, lipid metabolism

## Abstract

**Introduction:**

The schweinfurthin class of compounds has been under investigation for two decades as potential cancer treatment candidates. Recent work demonstrating that these agents can induce immunogenic cell death has led to increased interest in our lab in establishing a development candidate.

**Methods:**

Here we use microsomal stability studies and reaction profiling of CYP450s involved in the metabolism of the two compounds 5’-methylschweinfurthin G (MeSG, TTI-3114) and TTI-4242, as well as CYP450 induction using cell culture in HepaRG cells.

**Results:**

TTI-4242 is more stable to microsomal enzymes than MeSG. CYP3A4 is primarily responsible, with some contributions from CYP2D6, CYP2C9, CYP2C19, and CYP1A2. In addition, both compounds induce the activity of CYP2D6.

**Discussion:**

Here we set out to determine the initial metabolic disposition of two of the most advanced schweinfurthin compounds, focusing on reaction profiling and cytochrome P450 (CYP450) induction, to help choose which of these should have more resources devoted to chemical synthesis and *in vivo* testing.

**Conclusion:**

The results from this study have led us to focus our resources on the indole-containing schweinfurthin analog TTI-4242 for further development as a treatment for melanoma and other cancers.

## 1 INTRODUCTION

The schweinfurthin family of natural products was discovered in the late 20^th^ century by the National Cancer Institute’s (NCI) Developmental Therapeutics Program [1]. These natural products demonstrated potent and selective activity in the NCI-60 human cell line panel [2, 3]. Particularly strong activity was noted in the CNS panel of cell lines, as well as in some melanoma and renal cancer lines. Ovarian and lung cancer cell lines proved highly resistant to the compounds, and the overall pattern of activity was not related to any clinically used compounds, indicating the potential for a novel mechanism of action [4].

A drug discovery effort was initiated around these compounds by the Wiemer group at the University of Iowa [5] and later by a small company, Terpenoid Therapeutics Inc., of Coralville, Iowa. These efforts led to the synthesis of numerous compounds, which have been the subject of several studies aimed at elucidating the mechanism of action and developing a drug based on the scaffold. The totality of this previous work has been reviewed [6].

Concurrent with these synthetic studies, numerous groups have been working to elucidate the target or basis of this anticancer activity. We showed that the fluorescent probe and 3-deoxyschweinfurthin B (3-dSB, a first-generation synthetic analog) likely caused apoptosis *via* PARP cleavage, which could be blocked by caspase 9 inhibition at 36 hours of treatment [6].

Our group has been interested in the isoprenoid biosynthetic pathway (IBP) for over two decades, and because the schweinfurthins have structural similarities to isoprenoids, we hypothesized that they may affect the IBP [7]. Indeed, we found that co-treatment with 3-dSB and lovastatin was synergistic for growth inhibition. In contrast, inhibitors of other enzymes in the IBP did not show a synergistic effect and, in some cell lines, were antagonistic to the growth inhibition effects [7]. Schweinfurthin treatment reduced the cellular levels of farnesyl and geranylgeranyl pyrophosphate, key intermediates in the IBP, in both established cell lines and primary patient acute leukemia isolates [7]. All of these effects were more pronounced in lipid-depleted media. Interestingly, upon testing the message levels of IBP-related genes, we found that treatment with 3-dSB reversed the effects of lovastatin treatment.

Our next series of experiments aimed more directly at the sterol regulatory pathways. In this study, we found that 3-dSB lowers cholesterol levels by dramatically reducing the synthesis of cholesterol while simultaneously increasing cholesterol efflux through elevated levels of the transport protein ATP-binding cassette subfamily A member 1 (ABCA1). Our data indicated that cholesterol efflux by ABCA1, which typically relies on delivery to apolipoprotein A1, was independent of this mechanism [6]. This led to the hypothesis that schweinfurthins could be liver X receptor (LXR) agonists. LXR acts in opposition to the sterol regulatory element-binding proteins (SREBP). These two signaling systems broadly control sterol levels in cells, with SREBP acting to increase sterol levels in response to low membrane cholesterol in the ER and LXR acting to decrease sterol levels [8]. Interestingly, testing 3-dSB for activation of both isoforms of LXR (α and β) indicated that this compound does not act through this mechanism [9].

The work of Burgett *et al*. [10], who assayed the schweinfurthins as well as other compounds (OSW-1, cephalostatins, *etc*.) with similar NCI-60 profiles, showed that the compounds bind the protein oxysterol-binding protein (OSBP) as well as the related protein ORP4L, another member of the oxysterol-binding protein family (ORPs). In 2015, Bao *et al*. demonstrated that the schweinfurthins rapidly internalize into endosomes/lysosomes and then cause perturbation and dispersal of the trans-Golgi network [11]. This team also expanded the range of ORPs known to bind the compounds, showing that a biotinylated schweinfurthin G (SG) derivative binds to ORP1S and ORP1L with an affinity of around 200 nM. In addition, they showed that SG increased the affinity of OSBP for one of its ligands, phosphatidylinositol 4-phosphate (PI4P).

A more recent report by a group at the NCI used a new bioinformatics tool called CellMinerCDB, which allows interactive queries across several large datasets, including the NCI-60, NCI-SCLC, Sanger/MGH GDSC, and the Broad CCLE/CTRP, to examine the activity of schweinfurthin A and MeSG [12]. This group submitted these two schweinfurthins to the Sanger/Mass General Hospital Genomics Drug Sensitivity in Cancer Database for growth inhibition assessment across 585 cell lines. They found SA and MeSG to be highly correlated in activity, which, along with our own extensive NCI-60 assay data and RNAseq analysis, reinforces the notion that all these compounds work through a specific mechanism. These studies point to ORP signaling and lipid homeostasis as key to the Schweinfurthin effects and, taken together, imply that several ORPs beyond OSBP could be directly involved in aspects of the compound’s activity.

As part of these ongoing studies, we have very recently discovered that the schweinfurthin analogs TTI-3114 (also called 5’-methylschweinfurthin G or MeSG) and TTI-4242 (Fig. **1**) are potent inducers of a form of immunogenic cell death (ICD) [13]. ICD, which is downstream of several programmed cell death pathways [14], can lead to immune-mediated anticancer responses [15]. We have demonstrated that treatment with either of these agents leads to potent enhancement of response to anti-PD-1 antibody treatment in a mouse model of melanoma. In this study, approximately 30% of the mice treated with the combination had durable tumor immunity and did not develop tumors upon re-challenge, indicating they were essentially cured [13]. This effect was observed only in mice with intact immune systems and at safe doses of the schweinfurthin compounds.

Since the ICD in melanoma publication, we have been studying the mechanism of ICD induction using the compound TTI-4242. Interestingly, this compound induces ICD in a manner that is somewhat different from the more traditional compounds known to have these effects, such as mitoxantrone, oxaliplatin, or ionizing radiation [16]. These studies show that treatment with MeSG causes cell death at very low concentrations in some cell lines, while others are more resistant but do eventually succumb to apoptosis correlated with mitochondrial membrane potential disruption. It is possible that this effect represents a compensatory mechanism allowing survival from the initial effects of lower doses of the compound. The next steps in our research require the synthesis of large amounts of the compound to advance the studies into animal models of several cancers.

## 2 MATERIALS AND METHODS

### 2.1 Microsomal Stability and Reaction Profiling

#### 2.1.1 Materials

NADPH was obtained from Santa Cruz Biotechnology (Dallas, TX). Human liver microsomes (mixed-sex pooled) were obtained from Sekisui Xenotech (Kansas City, Kansas). Furafylline, sulfaphenazole, 3-benzylnirvanol, quinidine, and ketoconazole were all obtained from Sigma Aldrich (St. Louis, MO).

#### 2.1.2 Methods

All assays were performed in triplicate at 37°C. All samples contained 40 M of the test compound, human liver microsomes (0.5 mg/mL protein), NADPH (1 mM), phosphate buffer (100 mM NaPO4, 5 mM MgCl2, pH 7.5), and one specific CYP inhibitor. In samples containing furafylline, microsomes, NADPH, and phosphate buffer are added, and the mixture is pre-incubated for 15 minutes. The reaction is then initiated by adding the test compound. For all other CYP inhibitors, the inhibitor is added along with the test compound for a 5-minute pre-incubation, and the reaction is initiated by adding NADPH. A list of inhibitors and their incubation concentrations used is in Table **1**. These inhibitors were chosen based on literature indicating specificity for the indicated CYP isoforms [17].

Aliquots were taken at 0, 15, 30, 45, and 60 minutes and placed in acetonitrile to terminate the reaction. After being collected, samples are centrifuged at 20,000 x g for 5 minutes at 4°C. Samples were placed in HPLC vials and quantified by reverse-phase HPLC with fluorescence detection.

HPLC analysis was carried out on a Shimadzu Prominence system with a low-pressure binary gradient pump LC-20AD connected to an SPD-20AV fluorescence detector and a SIL-20AC autosampler. The sample analysis was performed on a Phenomenex C18 reversed-phase analytical column (250 × 3.0 mm, 5 μm) (Torrance, CA), protected by a Phenomenex Luna guard column. The mobile phase consisted of 20 mM ammonium acetate in 90% acetonitrile and 25 mM ammonium acetate in 40% acetonitrile (75:25 v/v), with a total run time of twenty minutes. The sample injection volume was 50 μL, and the column temperature was maintained at ambient conditions. MeSG was monitored by fluorescence detection at 330 nm (excitation) and 390 nm (emission), and TTI-4242 was detected at 345 nm (excitation) and 415 nm (emission).

The intrinsic clearance (microsomal clearance) was calculated from the slope of the natural logarithm of the parent drug peak area (HPLC), which provides the degradation rate constant for the reaction. The rate constant for substrate disappearance, combined with the amount of microsomal protein and the incubation volume, was then used to calculate the CL_mic_. The slope of the substrate concentration versus time was plotted in Prism (GraphPad), and the error in the slope calculation was defined as the 95% confidence interval of the best fit of the values.

### 2.2 CYP Induction

HepaRG cells (ThermoFisher; Waltham, MA) were cultured and differentiated as previously described [18, 19]. Near confluent 10 cm plates of differentiated HepaRG cells were treated with 0.5 µM MeSG, TTI-4242, or 20 µM rifampicin for 24 hours. RNA was isolated using trizol as previously described, and 1000 ng of RNA was reverse transcribed into cDNA (ThermoFisher; Waltham, MA). RT-PCR was performed using Taqman probes (ThermoFisher; Waltham, MA) for: CYP1A2 (Hs01070374_m1), CYP2B6 (Hs03044634_m1), CYP2C8 (Hs00258314_m1), CYP2C9 (Hs00426397_m1), CYP2C19 (Hs00426390_m1), CYP2D6 (Hs02576168_g1), CYP3A4 (Hs00430021_m1), and GAPDH (Hs99999905_m1).

## 3 RESULTS

### 3.1 Microsomal Stability of MeSG and TTI-4242

To begin distinguishing the compounds from each other, we carried out a microsomal stability profile for each. In human microsomes, TTI-4242 was more stable than MeSG, showing a much higher percentage of parent compound remaining at the 60-minute time point. The time-course data indicated that MeSG was a substrate for extensive microsomal enzyme activity (Fig. **2A** and **B**). Calculated microsomal clearance was also greater for MeSG, and the corresponding half-life was lower, indicating substantial metabolism of this compound by CYP enzymes compared to TTI-4242 (Table **2**).

To determine the primary CYP enzymes responsible for the phase 1 clearance of the schweinfurthin compounds, we performed reaction profiling with known inhibitors of five key CYP enzymes. Inhibition of all the CYPs tested showed effects on the metabolic clearance of both compounds (Table **3**). However, the most profound effect was with the inhibition of CYP3A4, which essentially completely stopped the microsomal degradation (half-life was increased by 122 times) of MeSG. This was true for TTI-4242 as well; however, the effect of CYP3A4 inhibition was not as profound. This indicates that CYP3A4 predominantly metabolizes both of these compounds.

### 3.2 Cytochrome P450 Induction by Schweinfurthin Treatment

Treatment of HepaRG cells with schwienfurthin compounds MeSG and TTI-4242 led to increased mRNA levels of CYP2D6. None of the other CYP message levels were changed (Fig. **3**). In our results, the positive control rifampicin showed good induction of the CYPs, except for CYP2D6, despite this CYP being the only one induced by the schweinfurthin compounds.

## 4 DISCUSSION

The schweinfurthin compounds are very potent inducers of immunogenic response, as evidenced by our experiments in murine melanoma. Both MeSG and TTI-4242 were effective in this regard; however, the maximum tolerated dosage of MeSG is lower, and therefore the dose tested (20 mg/kg) was lower than the dose of TTI-4242 (30 mg/kg). In order to decide on a clinical development candidate, we sought to utilize preclinical metabolic models to refine our knowledge of these two compounds [20]. The compounds were provided by Terpenoid Therapeutics, which until recently had been developing them for potential use in brain cancer. In addition, a group at the University of Iowa used MeSG in a mouse model of chondrosarcoma, showing some tumor reduction at the 20 mg/kg dose [6].

For parenteral delivery, the highly lipophilic compound MeSG required an enabling formulation using a proprietary ternary mixture of excipients. Terpenoid Therapeutics reported that the TTI-4242 compound was significantly more soluble than MeSG, but still required a similar formulation for our melanoma models. As part of earlier drug development efforts, CACO-2 permeability screens were carried out, and the TTI-4242 compound showed roughly twice the permeability (63 nm/sec *vs*. 33 nm/sec for MeSG), and neither of these compounds is a substrate for the P-glycoprotein (PgP) drug efflux pump [21].

Here, we sought to test the microsomal metabolism of these two compounds as a screen prior to initiating pharmacokinetic studies. Our ongoing studies in melanoma and PK analyses will require the synthesis of a relatively large amount of compound, and the synthesis of either of these is multi-step, thus necessitating an early choice between the two. The totality of the information outlined above was already favorable to the choice of TTI-4242 for further study. Testing both compounds for microsomal clearance indicated that MeSG is highly susceptible to CYP-mediated metabolic transformation, with a half-life of 19 minutes. Reaction profiling with specific CYP inhibitors showed that the main metabolizing enzyme for MeSG was CYP3A4. While CYP3A4 was also the main enzyme implicated in TTI-4242 metabolism, the extent to which metabolism was reduced with CYP3A4 inhibition was considerably less. MeSG was also metabolized to a significant extent by CYP2D6 and CYP2C9, whereas TTI-4242 showed metabolism by CYP2C19 and CYP1A2.

While there is a large dependence on CYP3A4 for the metabolism of both TTI-3114 and TTI-4242, the overall metabolism of TTI-4242, including *via* CYP3A4, is much less. This suggests that rapid oxidation at a position on the phenolic ring is prevented by the conversion to the indole substructure in TTI-4242. Since CYP3A4 is responsible for the metabolism of approximately 30% of approved drugs [22], this represents a potentially serious liability for TTI-3114 compared with TTI-4242. The potential for drug–drug interactions and the need for exclusion criteria in clinical development would be high. A limitation of this analysis is that the reduction of metabolism by ketoconazole, the CYP3A4 inhibitor, is so extensive that the percent metabolized is reduced to essentially nil. Although a slight difference could be observed and microsomal clearance calculated from the very small amount metabolized, the key takeaway is that TTI-3114 is rapidly cleared, predominantly by CYP3A4. The fact that in our preliminary results in murine melanoma models TTI-3114 performed similarly to TTI-4242 suggests that the CYP3A4 metabolite may also be active, which would significantly increase the resources required for IND-enabling studies. Taken together, these considerations strongly support TTI-4242 as the preferred development candidate.

While the pronounced effects of CYP3A4 inhibition on TTI-3114 are clear (~120-fold increase in projected t1/2_{1/2}1/2), the effects of inhibition of the other CYPs tested are less dramatic. Taken together, CYP2D6 (20%), CYP2C9 (13%), CYP2C19 (7%), and CYP1A2 (9%) account for primary metabolism of an additional 49% of approved drugs. TTI-4242, in contrast, shows relatively balanced metabolism among these enzymes. Inhibition of these enzymes has effects on TTI-4242 metabolism ranging from minimal for CYP2D6 and CYP2C9 to moderate for CYP2C19 (3-fold) and CYP1A2 (2.5-fold).

## CONCLUSION

Here, we describe initial metabolic stability studies on two potential schweinfurthin analogs under consideration for development as anticancer agents. The early-stage compound TTI-3114 (MeSG) is highly susceptible to microsomal CYP oxidation, resulting in low availability. Conversion of the phenolic oxygen into an indole substructure, as in TTI-4242, leads to enhanced microsomal stability while preserving activity, supporting the choice of this compound for further development. In addition, toxicities observed in prior *in vivo* studies indicate that both compounds have similar toxicity profiles, with TTI-3114 being less well tolerated than TTI-4242. We will conduct rat pharmacokinetic studies as the next phase of development for TTI-4242 to evaluate compound exposure and inform IND-enabling studies.

## AUTHORS’ CONTRIBUTIONS

The authors confirm their contributions to the paper as follows: JN conceived of and coordinated the experiments and results, and drafted the manuscript. JR and WRK planned and carried out experiments, drafted the results, and drafted and reviewed the manuscript.

## Figures and Tables

**Fig. (1):**
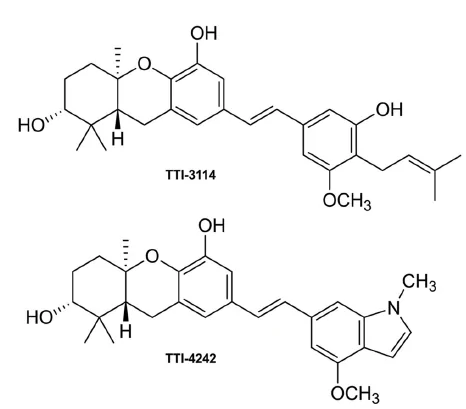
Structures of MeSG and TTI-4242.

**Fig. (2):**
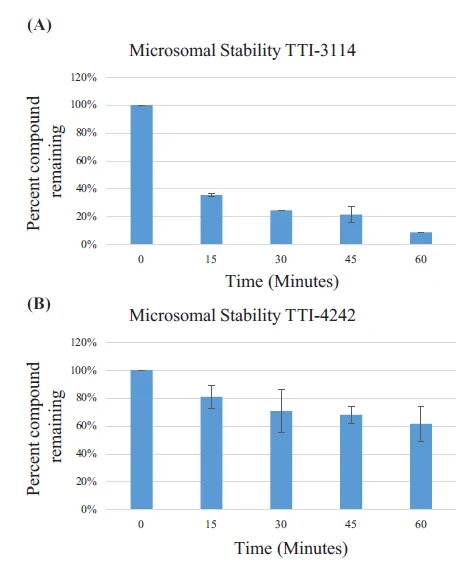
Microsomal stability time course showing percent parent compound remaining for schweinfurthin analogs **A**) MeSG and **B**) TTI-4242.

**Fig. (3):**
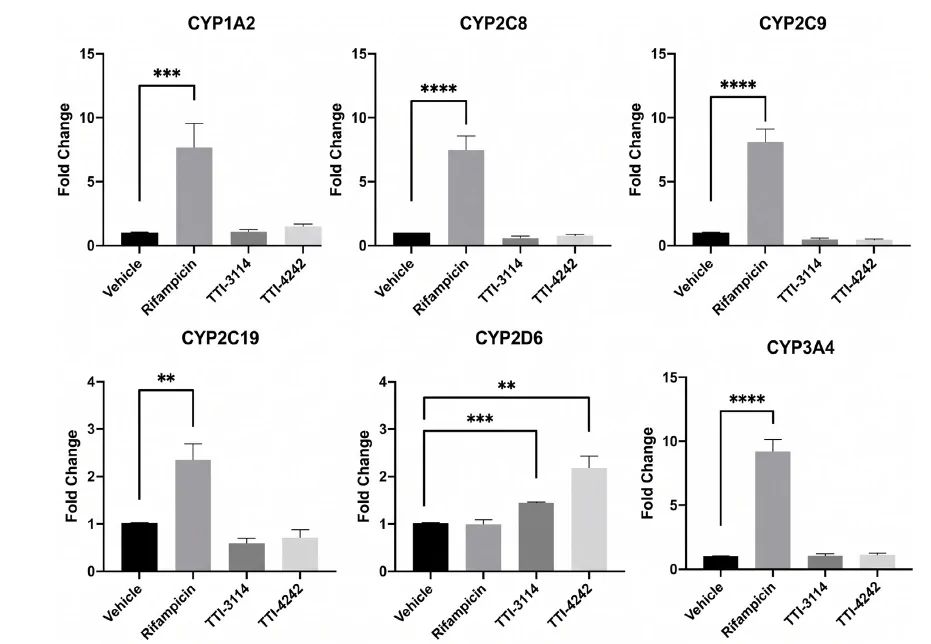
mRNA levels showing individual CYP enzyme induction by schweinfurthins TTI-3114 (MeSG) and TTI-4242 after 24 hour incubation. ** *p*<0.01, *** *p*<0.005, **** *p*<0.001.

**Table 1:** CYP inhibitors and concentrations used.

**CYP**	**Inhibitor**	**Incubation Concentration**
CYPsC19	3-Benzylnirvanol	20 μM
CYP1A1/2	Furafylline	80 μM
CYP3A4	Ketoconazole	80 μM
CYP2D6	Quinidine	20 μM
CYP2C9	Sulfaphenazole	80 μM

**Table 2:** Calculated microsomal clearance kinetics.

**Compound**	**Half-life (t_1/2_) min**	**Microsomal Clearance (CL_mic_) mL/mg/min**
MeSG	19	79
TTI-4242	81	18

**Table 3:** Changes in microsomal clearance kinetics with CYP inhibition.

**CYP**	**Inhibitor**	**Percent Metabolized 60 mins**	**TTI-3114**		**Percent Metabolized 60 mins**	**TTI-4242**	
			**Half-life (t_1/2_) min**	**Microsomal Clearance (CL_mic_) μL/mg/min**		**Half-life (t_1/2_) min**	**Microsomal Clearance (CL_mic_) μL/mg/min**
** **	**None**	66	NA	NA	58	NA	NA
CYPC19	3-Benzylnirvanol	37	92	16.8	13	243	6.35
CYP1A1/2	Furafylline	52	67	22.8	17	200	7.72
CYP3A4	Ketoconazole	<0.1	2330	0.66	<0.1	464	3.3
CYP2D6	Quinidine	50	166	9.3	58	121	12.8
CYP2C9	Sulfaphenazole	40	225	6.8	58	78.9	19.52

## Data Availability

The data generated and/or analyzed during the current study are available from the lead contact upon reasonable request. This paper does not report original code. All data generated and analyzed to support this manuscript are included herein. Please contact the corresponding author for any enquiries.

## LIST OF ABBREVIATIONS

NCI: National Cancer Institute
CNS: Central Nervous System
3-dSB: 3-Deoxyschweinfurthin B
PARP: Poly (ADP-ribose) Polymerase
IBP: Isoprenoid Biosynthetic Pathway
ABCA1: ABC-cassette subfamily A Member 1
LXR: Liver-X-Receptor
SREBP: Sterol Regulatory Element Binding Proteins
ER: Endoplasmic Reticulum
OSBP: Oxysterol Binding Protein
ORP: Oxysterol Binding Related Protein
NCI-SCLC: National Cancer Institute’s Small Cell Lung Cancer Database
Sanger/MGH GDSC: Sanger Institute/Massachusetts General Hospital Genomics of Drug Sensitivity in Cancer
Broad CCLE/CTRP: Broad Institute Cancer Therapeutics Response dataset
SA: Schweinfurthin A
MeSG: 5’-Methylschweinfurth G
ICD: Immunogenic Cell Death
PD-1: Programmed Cell Death – 1 Protein
NADPH: Nicotinamide Adenine Dinucleotide Phosphate
CYP: Cytochrome P450 Enzymes
HPLC: High-Performance Liquid Chromatography
